# Black/White Disparities in Obesity Widen with Increasing Rurality: Evidence from a National Survey

**DOI:** 10.1089/heq.2021.0149

**Published:** 2022-03-03

**Authors:** Steven A. Cohen, Caitlin C. Nash, Erin N. Byrne, Lauren E. Mitchell, Mary L. Greaney

**Affiliations:** Department of Health Studies, College of Health Sciences, University of Rhode Island, Kingston, Rhode Island, USA.

**Keywords:** racial disparities, obesity, diabetes, rural health

## Abstract

**Background:**

Racial health disparities in obesity and obesity-related conditions and behaviors are well documented, although a small body of research suggests that geographic factors (e.g., socioeconomic status [SES] and rural/urban status) may alter the magnitude of these disparities.

**Methods:**

This study explored how rurality moderates black/white health disparities using a nationally representative sample from the 2012 Behavioral Risk Factor Surveillance System (*n*=359,157). Respondents' county of residence was linked to the U.S. Census information to obtain the county-level Index of Relative Rurality (IRR). Weighted logistic regression was used to model obesity, diabetes, and lack of physical activity (PA) on race (black/white), IRR, and an interaction term of race and IRR, including covariates (age, sex, education, marital status, employment, and income).

**Results:**

Blacks were significantly more likely to have obesity, diabetes, and a lack of PA compared with whites. Irrespective of race, rural respondents were significantly more likely to have obesity (odds ratio [OR] 1.035, confidence interval [95% CI] 1.028–1.043) and a lack of PA (OR 1.045, 95% CI 1.038–1.053) than respondents in more urban areas. For obesity and diabetes, the interaction term for black×IRR quintile was significant and positive, indicating an increase in the magnitude of the black/white disparity with increasing rurality.

**Discussion:**

These findings underscore the need for policies and programs aimed to reduce racial disparities in obesity and related conditions to consider the geographic context in which these outcomes occur.

## Introduction

Racial disparities in health status, defined as differences in health status precipitated by social and economic disadvantage and/or systemic racism,^1^ are pervasive and well documented. Although life expectancy has increased across the United States over the past several decades,^1,2^ black/white disparities persist, with life expectancy among the black population being ∼4 years lower than the white population.^3^ Black/white disparities exist in all aspects of health, including health status, behaviors, and health care access, including cancer,^4,5^ chronic conditions,^6^ disability,^7–9^ maternal and child health,^10–12^ and access to health services.^13^ Furthermore, black and other minoritized populations have been disproportionately affected by recent acute declines in life expectancy due to COVID-19.^14^

The importance of rural/urban health disparities is increasingly being recognized as a critical issue in population health. Rural/urban health disparities include differences in health care, health behaviors, and health status. Residents in rural areas have reduced access to health care services and higher quality care than people in urban areas.^15,16^ Rural populations are less physically active,^16^ have higher rates of type 2 diabetes,^17^ obesity,^18^ poorer overall general health,^19^ lower health-related quality of life,^20,21^ and increased mortality^22,23^ than urban populations.

Rural/urban health disparities may be partially explained by socioeconomic, demographic, and environmental factors that underlie the cultural fabric of rural areas.^24^ These trends contribute to the increasing recognition that place of residence impacts one's health,^25,26^ but the mechanisms causing the disparities are less well understood.

Obesity is one of the most significant public health concerns affecting the United States and the global population. It is a risk factor for numerous adverse health outcomes, comorbidities, and chronic diseases across the life span,^27^ including hypertension,^28,29^ hyperlipidemia,^28,30^ other cardiovascular diseases independent of hypertension,^31,32^ and mortality.^33^

The prevalence of obesity has been steadily increasing in the United States. More than 40% of U.S. adults currently have obesity, an increase of 27% for women and 56% for men since 1999.^34^ Furthermore, each year, obesity and obesity-related conditions cost the U.S. health care system nearly $150 billion.^35^ Although not all type 2 diabetes-associated costs are directly attributable to obesity, direct health care for type 2 diabetes and related services exceeds $200 billion.^36^

There are well-established black/white disparities in obesity and related conditions such as type 2 diabetes.^6,37–40^ In 2018, the prevalence of obesity among non-Hispanic black women (57%) was substantially higher than that among non-Hispanic white women (40%). However, the prevalence in both groups increased significantly during the previous 20 years.^34^

Moreover, rural/urban disparities in obesity and obesity-related diseases are a public health issue in the United States.^17,18,41^ A 2015 study found that obesity prevalence was 36% higher in rural areas than in the urban areas of the United States, and specific socioeconomic, cultural, and environmental factors underlying these disparities were not identified.^42^ An earlier U.S. study also found a similar prevalence of obesity in rural areas (39.6%), nearly 20% higher than that identified in urban areas (33.4%). This difference was significant even after controlling for demographic, dietary, and physical activity (PA) variables.^43^

This study also indicated that the black/white difference in obesity prevalence was 30% higher in rural areas than in the urban areas, and this difference was significant even after controlling for other socioeconomic and demographic factors. Another study examining racial disparities in a major U.S. city found that the magnitude of black/white health disparities decreased once the analysis controlled for the social environment, suggesting that place, not race, is driving health disparities.^44^ This study did not, however, directly compare urban and rural areas.

An intersectionality framework may be useful to research and understand how multiple social structures jointly contribute to health disparities. Intersectionality is defined as a “theoretical framework for understanding how multiple social identities such as race, gender, sexual orientation, SES, and disability intersect at the micro-level of individual experience to reflect interlocking systems of privilege and oppression (i.e., racism, sexism, heterosexism, classism) at the macro social-structural level.”^45^

Using an intersectionality framework to research health disparities will allow for detecting multilevel health disparities. Intersectionality relates closely to the larger issue of social justice inherent in the broader field of public health research and advocacy.^45,46^ While intersectionality most often is used to assess joint effects of individual social constructs such as race and gender, there is increasing evidence that place-based characteristics such as rural/urban status may play a key role in promoting or mitigating other health disparities.^47^

Although some studies have explored the variability of black/white health disparities by geography, few have examined black/white health disparities in obesity and related conditions and behaviors by rural/urban status. Much of the previous research examining these disparities has focused separately on race/ethnicity or rural/urban status.^34,37–43^

Furthermore, programs and interventions designed to address racial/ethnic health disparities are often based on models developed in urban populations and applied to all populations and do not generally consider geographic differences.^48^ Therefore, there is a vital need to identify geographic variability in racial health disparities.^49^ This information is essential for developing evidence-based policies to reduce health disparities and improve overall population health. Therefore, the objective of this study was to use an intersectional framework to assess the potential for the moderation of black/white disparities in obesity, diabetes, and PA by rural/urban status.

## Methods

### Data set and sample

This is a secondary analysis of data from the 2012 Behavioral Risk Factor Surveillance System (BRFSS), the largest system of health-related telephone surveys administered by the Centers for Disease Control and Prevention (CDC).^50^ The BRFSS collects data annually from U.S. residents aged 18+ in all 50 states regarding their demographics, self-reported health-related risk behaviors, height, weight, chronic health conditions, and use of preventive services. Federal and state authorities use the BRFSS for planning and prevention efforts.^46^ Between 400,000 and 500,000 interviews with BRFSS respondents are conducted each year, with a total sample of 475,687 respondents in 2012. Response rates for landline and cell phones were 49.1% and 35.3%, respectively.^51^

The 2012 BRFSS sample was selected for this analysis as it was the last sample year in which the respondent's county of residence is available in the public-use database. Each of these respondents was linked to area-level data from the 2010 U.S. Census via the county Federal Information Processing Standard code.

### Outcome measures

#### Obesity status

Respondents' self-reported height and weight were used to calculate the body mass index (BMI), which was used to determine obesity status. Respondents whose BMI was 30 kg/m^2^ or above were classified as having obesity, while those whose BMI was below 30 kg/m^2^ were classified as not having obesity. Respondents with a BMI of <18 kg/m^2^ were excluded from the analysis (0.2%).^52^

#### Diabetes status

Self-reported diabetes status also was obtained from BRFSS respondents using the question, “Has a doctor, nurse, or other health professional ever told you that you had diabetes?” Respondents who answered “yes” were classified as having diabetes, while those answering “no” or “yes, but told during pregnancy,” were classified as not having diabetes.

#### PA participation

BRFSS respondents were asked one question about PA participation: “During the past month, other than your regular job, did you participate in any physical activities or exercises such as running, calisthenics, golf, gardening, or walking for exercise?” Respondents responded either “yes,” “no,” “don't know/not sure,” or could refuse to answer. Those who answered “don't know/not sure” or who refused to answer were coded as missing, and all others were coded as a binary measure (yes, no) of participation in PA in the past month. PA participation was reverse coded for analysis so that all “yes” responses for the three outcome measures (obesity, diabetes, and PA) referred to poorer health or behavioral outcomes. Therefore, this PA measure used refers to a lack of PA in the previous 30 days.

### Predictor measures

#### Race

Respondents were asked, “Which one of these groups would you say best represents your race?” Eight options were available: white, black, Asian, Native Hawaiian or other Pacific Islander, American Indian or Alaska Native, other, don't know, and multiracial. Of the total sample (*n*=475,687), the sample was restricted to respondents who reported being of either black or white race to align with the purpose of this study (*n*=359,157).

#### Rural/urban status

Rural/urban status or “rurality” was determined by linking each respondent's county of residence from the BRFSS data set to the corresponding information from the 2010 U.S. Census. The Index of Relative Rurality (IRR) at the county level,^53^ the central measure of rural/urban status used in this analysis, was derived from four measures at the county level: percent urban population, population size, population density, and the distance to the nearest metropolitan area. County-level IRR was categorized into quintiles for analysis, with the first quintile (Q1) being the most rural and Q5 being the most urban.

#### Covariates

Other exposure variables used included sex (male, female), as ascertained through telephone interview, age category (18–24, 25–34, 35–44, 45–54, 55–64, 65+), marital status (currently married, not currently married), education (less than bachelor's degree, bachelor's degree or higher), current employment status (yes, no), and annual household income category (<$25,000, $25,000–49,999, $50,000+, and missing).

### Data analysis

The analytic sample included respondents with complete data on race (black or white) and IRR (*n*=359,157, 75.5% of the entire sample). Descriptive statistics, including frequencies for categorical and ordinal variables and means and standard deviations for discrete and continuous variables, were obtained for all the study's exposure and outcome measures. Chi-squared tests were used to assess bivariate associations between pairs of categorical variables. One-way analysis of variance was used to assess the associations between categorical and continuous variables, and Pearson or Spearman rank correlations were used to estimate correlations between pairs of discrete or continuous variables. Trend tests also were examined to assess associations between each IRR quintile and each outcome.

Three sets of generalized linear models with a logistic link were used to measure the multivariable associations between the exposure measures (race and rural/urban status) and the three outcome measures. Three separate models for obesity, diabetes, and lack of PA were analyzed. The first set of multivariable models considered the IRR quintile as an ordinal variable and included main effects for both race, IRR quintile, and interaction terms for race×IRR quintile.

The second set of multivariable models, stratified the sample by IRR quintile, examined the adjusted associations between race and each of the outcomes within every IRR quintile. The third set of models considered each subgroup (race×IRR quintile) as a distinct subgroup and modeled each of the three outcomes on these subgroups using whites in the most urban IRR quintile as the reference group.

All models included the study covariates (sex, age group, marital status, education, employment status, and income) and used the BRFSS-provided sample weights in the analysis to account for complex sampling. A sensitivity analysis using quintiles of Rural/Urban Continuum Codes (RUCC), a commonly-used measure of county-level rural/urban status,^54,55^ in place of the IRR in the models to determine if any of the observed associations with rural/urban status were dependent on the way in which it was measured. IBM SPSS version 27 (Armonk, NY) and SAS version 9.4 (Cary, NC) were used for all the analyses. Ethical approval was obtained to conduct this analysis (University of Rhode Island IRB approval number 1688526-1).

## Results

Descriptive statistics for the analytic sample (Table 1) show that the majority (57.7%) of respondents resided in counties in the most urban IRR quintile. Compared with respondents from the most rural counties for IRR quintile (Q1), respondents from the most urban counties (Q5) were more than twice as likely to be black, 83% more likely to have at least a bachelor's degree, 16% more likely to be employed, 58% more likely to be in the highest income category ($50,000+), and 12% less likely to be married.

**Table 1.** Descriptive Statistics for Analytic Sample, Overall and Stratified by Quintile of Index of Relative Rurality

		Quintile of IRR				
	Overall	1 (most rural)	2	3	4	5 (most urban)
*N* (%)	359,157	9843 (2.7)	31,852 (8.9)	43,408 (12.1)	66,769 (18.6)	207,285 (57.7)
Race						
White	322,266 (84.7)	9264 (91.6)	30,322 (92.8)	40,481 (91.5)	62,664 (91.8)	179,535 (81.6)
Black	36,891 (15.3)	579 (8.4)	1530 (7.2)	2927 (8.5)	4105 (8.2)	27,750 (18.4)
Sex						
Male	142,359 (47.6)	3894 (47.6)	12,520 (48.7)	16,781 (47.6)	26,680 (48.7)	82,484 (47.9)
Female	216,798 (52.4)	5949 (52.4)	19,332 (51.3)	26,627 (52.4)	40,089 (51.3)	124,801 (52.1)
Age						
18–24	15,074 (11.3)	308 (9.2)	1064 (9.8)	1626 (11.1)	2711 (11.4)	9365 (11.4)
25–34	30,354 (14.3)	687 (12.0)	2357 (13.8)	3245 (14.0)	5401 (13.8)	18,664 (14.5)
35–44	42,545 (15.6)	984 (12.8)	3227 (14.1)	4431 (14.3)	7766 (15.5)	26,137 (16.0)
45–54	63,905 (19.6)	1668 (18.5)	5362 (19.2)	7607 (19.1)	11,577 (19.1)	37,691 (19.8)
55–64	83,960 (18.0)	2490 (21.2)	7868 (19.3)	10,463 (18.7)	15,692 (18.2)	47,447 (17.7)
65+	123,319 (21.3)	3706 (26.3)	11,974 (23.8)	16,036 (22.7)	23,622 (22)	67,981 (20.7)
Mean (SD)	47.2 (18.2)	50.4 (18.3)	49.2 (18.5)	48.8 (18.4)	48.2 (18.3)	46.6 (18.2)
Bachelor's degree						
No	228,479 (73.0)	7158 (83.3)	22,702 (83.5)	31,508 (83.4)	45,679 (79.7)	121,432 (69.4)
Yes	130,079 (27.0)	2664 (16.7)	9102 (16.5)	11,829 (16.6)	20,978 (20.3)	85,506 (30.6)
Currently employed						
No	182,315 (44.9)	5458 (51.7)	16,921 (48.4)	23,408 (48.6)	34,915 (46.4)	101,613 (43.8)
Yes	175,860 (55.1)	4357 (48.3)	14,850 (51.6)	19,882 (51.4)	31,696 (53.6)	105,075 (56.2)
Currently married						
No	167,270 (47.9)	4207 (42.5)	13,761 (43.1)	19,111 (44.7)	28,936 (44.1)	101,255 (49.6)
Yes	190,515 (52.1)	5613 (57.5)	17,989 (56.9)	24,192 (55.3)	37,630 (55.9)	105,091 (50.4)
Annual income						
<$25k	86,849 (23.7)	3108 (32.5)	9161 (30.9)	12,504 (29.9)	16,707 (25.5)	45,369 (21.9)
$25–49k	81,612 (21.9)	2516 (26.6)	8005 (25.0)	10,834 (24.2)	15,969 (24.2)	44,288 (20.8)
$50+	143,303 (41.7)	2956 (28.5)	10,450 (31.0)	14,082 (31.5)	25,052 (37.1)	90,763 (45.0)
N/A	47,393 (12.6)	1263 (12.4)	4236 (13.1)	5988 (14.4)	9041 (13.2)	26,865 (12.3)
Obesity						
No	261,778 (72.7)	7016 (69.1)	22,958 (69.9)	30,688 (70.1)	47,766 (70.5)	153,350 (73.8)
Yes	97,379 (27.3)	2827 (30.9)	8894 (30.1)	12,720 (29.9)	19,003 (29.5)	53,935 (26.2)
Diabetes						
No	313,797 (89.8)	8475 (88.2)	27,659 (88.2)	37,472 (88.1)	57,955 (89.1)	182,236 (90.2)
Yes	44,860 (10.2)	1356 (11.8)	4146 (11.8)	5880 (11.9)	8714 (10.9)	24,764 (9.8)
PA						
No	271,617 (77.6)	7059 (71.7)	23,263 (72.5)	31,119 (72.2)	49,491 (75.3)	160,685 (79.2)
Yes	86,710 (22.4)	2764 (28.3)	8539 (27.5)	12,220 (27.8)	17,153 (24.7)	46,034 (20.8)

IRR, Index of Relative Rurality; PA, physical activity.

Respondents in rural areas tended to be younger than those living in more urban areas. Having obesity, diabetes, and a lack of PA were more common among respondents in rural areas than in urban areas. Trend tests by IRR quintile were significant for all the examined variables (*p*<0.001 for all).

Black respondents were more likely than white respondents to have obesity, diabetes, and lack of PA (79.6%, 94.5%, and 28.4%, respectively; *p*<0.001) (Table 2). Regardless of race, those living in rural areas were more likely to have obesity (odds ratio [OR] 1.035, confidence interval [95% CI] 1.028–1.043) and a lack of PA (OR 1.045, 95% CI 1.038–1.053) than respondents in more urban areas. The interaction term for black×IRR quintile was significant and greater than the one for having obesity and diabetes, indicating a significant increase in the association between race and each outcome with increasing rurality.

**Table 2.** Adjusted Odds Ratios of Obesity, Diabetes, and Lack of Physical Activity for Race (Black vs. White), Rural/Urban Status, and the Interaction of Race and Rural/Urban Status

	Obesity	Diabetes	Lack of PA
Overall model			
Black (vs. white)	**1.796 (1.749–1.844)**	**1.945 (1.879–2.014)**	**1.284 (1.248–1.321)**
Rural/urban quintile	**1.035 (1.028–1.043)**	1.005 (0.995–1.014)	**1.045 (1.038–1.053)**
Black×rural/urban quintile	**1.055 (1.030–1.080)**	**1.044 (1.014–1.075)**	1.018 (0.993–1.044)

Boldface indicates statistical significance at *p* < 0.05.

Adjusted for sex, marital status, education, employment, income, and age group.

Similar results were observed in the models stratified by the IRR quintile that examined the direct association between race and the three outcome measures (Table 3). In these models, black respondents were more likely than white respondents to have obesity, diabetes, and a lack of PA regardless of the rural/urban status. However, the strength of the association was generally higher in more rural areas than in the urban areas (*p*<0.001 for trend), with the strongest associations for all three outcomes observed in Q2, the second-most rural quintile. In the sensitivity analysis using RUCC in place of IRR in the models, the results showed identical patterns of statistical significance.

**Table 3.** Adjusted Odds Ratios of Obesity, Diabetes, and Lack of Physical Activity for Race (Black vs. White), Stratified by Quintile of Index of Relative Rurality

	Obesity	Diabetes	Lack of PA
Black (vs. white) in models stratified by IRR quintile			
Q1 (most rural)	2.034 (1.709–2.421)	2.122 (1.717–2.621)	1.273 (1.061–1.528)
Q2	2.222 (1.998–2.742)	2.350 (2.067–2.673)	1.447 (1.296–1.617)
Q3	1.889 (1.748–2.043)	1.910 (1.732–2.107)	1.291 (1.189–1.401)
Q4	1.908 (1.787–2.038)	2.016 (1.854–2.193)	1.289 (1.201–1.384)
Q5 (most urban)	1.828 (1.778–1.880)	1.996 (1.924–2.071)	1.302 (1.263–1.343)

Adjusted for sex, marital status, education, employment, income, and age group.

Table 4 shows the estimated ORs from the final set of multivariable models in which each outcome was modeled using the entire analytic sample and indicator variables for each race×IRR quintile group with, as previously noted, white respondents from the most urban IRR quintile being the reference group. Black respondents had significantly higher likelihoods of all three outcomes across all five IRR quintiles. White respondents in IRR quintiles Q1–Q4 (all quintiles except the most urban) were significantly more likely to have obesity and a lack of PA. For diabetes, the association was significant among white respondents in IRR quintiles Q3 and Q4 only.

**Table 4.** Adjusted Odds Ratios of Obesity, Diabetes, and Lack of Physical Activity by Race (Black vs. White) and Quintile of Index of Relative Rurality Compared with Urban Whites

IRR quintile					
Has obesity	1	2	3	4	5
White	**1.10 (1.05–1.15)**	**1.07 (1.04–1.10)**	**1.15 (1.12–1.18)**	**1.13 (1.11–1.15)**	1 (Ref.)
Black	**2.33 (1.97–2.75)**	**2.43 (2.20–2.69)**	**2.16 (2.01–2.33)**	**2.15 (2.02–2.30)**	**1.82 (1.77–1.87)**
Has diabetes					
White	1.02 (0.95**–**1.08)	0.99 (0.95**–**1.03)	**1.04 (1.01–1.08)**	**1.06 (1.03–1.09)**	1 (Ref.)
Black	**2.28 (1.87–2.79)**	**2.36 (2.09–2.67)**	**2.06 (1.88–2.26)**	**2.16 (1.99–2.34)**	**1.96 (1.89–2.03)**
Lack of PA					
White	**1.14 (1.09–1.20)**	**1.10 (1.07–1.14)**	**1.18 (1.15–1.21)**	**1.12 (1.09–1.14)**	1 (Ref.)
Black	**1.44 (1.21–1.71)**	**1.60 (1.44–1.78)**	**1.52 (1.40–1.64)**	**1.45 (1.35–1.55)**	**1.30 (1.26–1.34)**

Boldface indicates statistical significance at *p* < 0.05.

Adjusted for sex, marital status, education, employment, income, and age group.

**FIG. 1.**
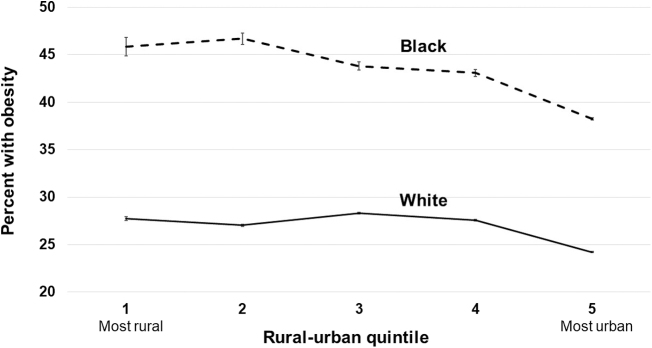
Adjusted prevalence of obesity in whites and blacks by quintile of Index of Relative Rurality (with 95% confidence intervals).

**FIG. 2.**
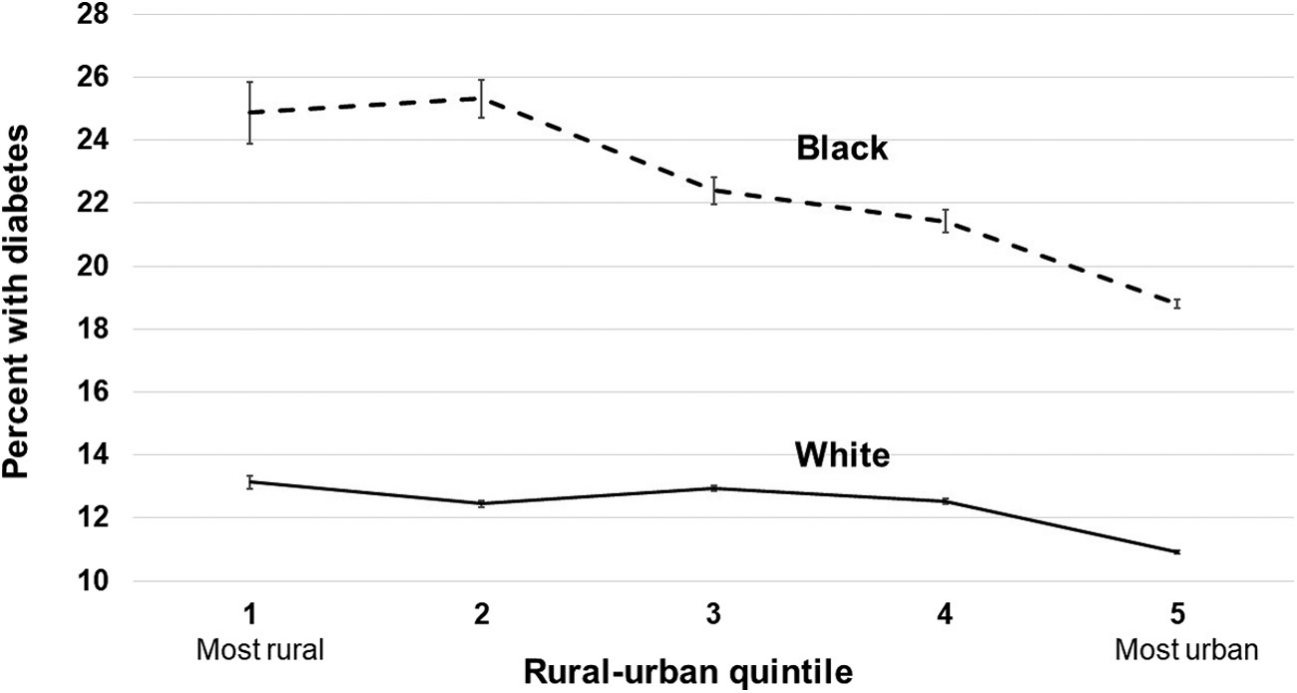
Adjusted prevalence of diabetes in whites and blacks by quintile of Index of Relative Rurality (with 95% confidence intervals).

**FIG. 3.**
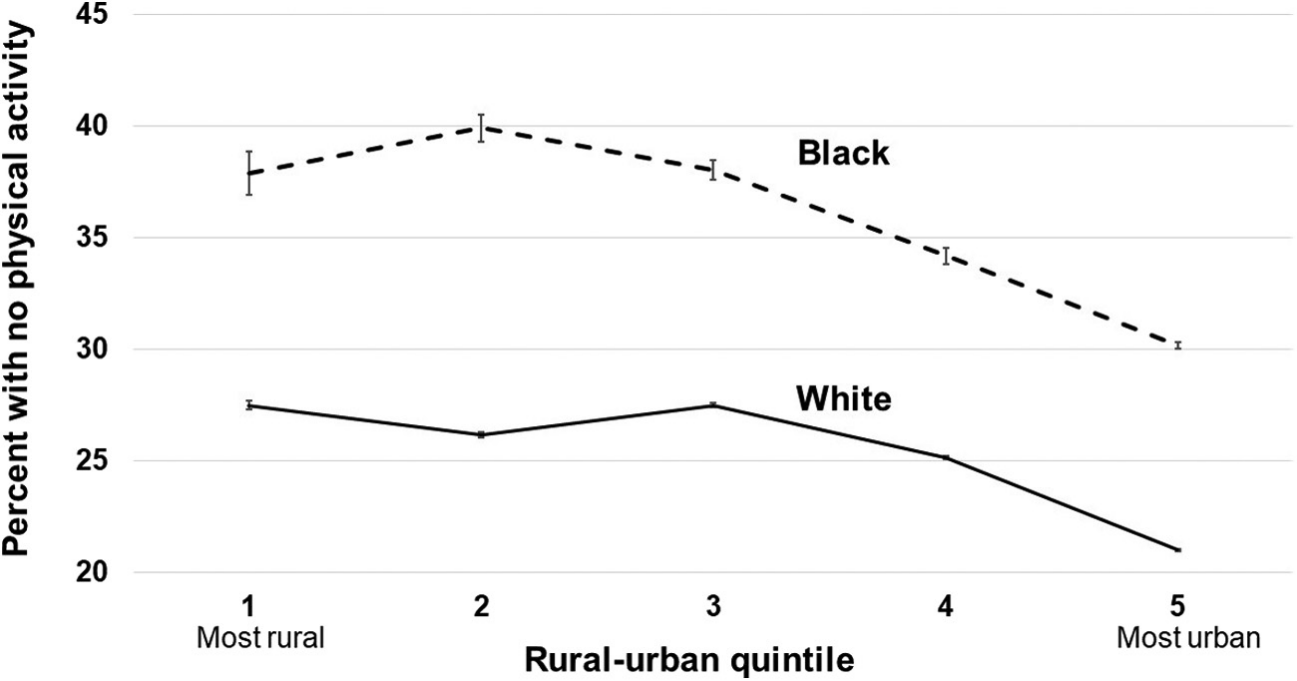
Adjusted prevalence of lack of physical activity in whites and blacks by quintile of Index of Relative Rurality (with 95% confidence intervals).

Model-predicted probabilities of each outcome by race and IRR quintile are illustrated in Figures 1–3. In the most rural areas (Q1), the predicted prevalence of obesity was 45.8% (95% CI 45.3–46.3) among blacks, and 27.7% (95% CI 27.6–27.8) among whites, an absolute difference of 18.1 percentage points. In the most urban areas (Q5), the predicted prevalence of obesity among blacks is 38.2% (95% CI 38.1–38.3) and among whites is 24.2% (95% CI 24.2–24.3), an absolute difference of 14.0 percentage points. The largest difference between blacks and whites for all three outcomes was observed in IRR quintile Q2, where the differences were 19.6, 12.9, and 13.7 percentage points for having obesity, diabetes, and a lack of PA, respectively.

## Discussion

Using an intersectional framework, this study assessed how rural/urban status potentially moderates the widely established associations between race and obesity and obesity-related conditions and PA. The prevalence of obesity and diabetes, and a lack of PA were significantly higher among black respondents than white respondents, irrespective of rural/urban status. The analysis determined that residents in rural areas have a higher prevalence of obesity and a lack of PA compared with those in urban areas, regardless of race.

However, the key study finding was that black/white differences in the prevalence of obesity and diabetes were significantly higher in rural than urban areas. Importantly, these trends were consistent regardless of rural/urban status measures (IRR vs. RUCC). Not only were the black/white disparities larger in rural areas, but the overall prevalence of obesity and diabetes was higher in rural areas for both white and black respondents as well.

These results are consistent with a recent study of mortality by race and rural/urban status that found that in nearly all the examined diseases and conditions, including cardiovascular disease, select cancers,^56^ and stroke, mortality rates were highest among black residents in rural areas.^57^ Another study found that rural black populations experience greater poverty and lack of access to care than other racial-geographic subgroups, potentially exposing this population to a greater risk of developing cancer and more severe cancer outcomes and lower chances of survival.^58^

Study findings have implications for research examining the intersections of race and place of residence. Although linear trends were observed with increasing rurality, a closer inspection of the data suggests that while rural populations overall experience higher levels of obesity and diabetes, the highest prevalence of each outcome, and the largest black/white disparities were observed in the counties of the second-highest rurality. The reasons for this are unclear. Nonetheless, these findings have critical implications for studying and addressing health disparities and improving population health.

Much of the prior research on rural/urban health disparities considers rural/urban status as a dichotomy,^59^ and using a dichotomous measure likely masks nuanced but important trends within the heterogeneity of what is considered “rural” using this dichotomy.^51,60,61^ While using a rural/urban dichotomous variable is convenient for analysis and interpretation, to understand nuanced nonlinear associations between rural/urban status and health outcomes, using an ordinal or continuous measure may be preferred.^62,63^

There is a vast body of literature on the numerous and complex causes and correlates of racial health disparities in the United States. These include, but are not limited to, structural racism,^64–66^ explicit or implicit discrimination and bias,^67–70^ culture,^68,71^ and geographic segregation.^13,72–74^ Addressing the root causes of racial disparities is a monumental and vitally important task that requires systematic, long-term, large-scale changes to nearly every aspect of U.S. society.^69,75^ The root causes of rural/urban health disparities are fairly well understood and generally point to socioeconomic, environmental, and cultural differences between the rural and urban areas.^24^ Examples include lower educational attainment,^76,77^ SES,^18,78,79^ access to and quality of health care services,^16,80,81^ and healthy behaviors in rural areas compared with urban areas.^16,82–84^

The direct psychosocial and biological pathways through which these factors directly impact population health and contribute to racial health disparities are less well understood. In addition, how other social constructs and place-based characteristics mitigate or enhance these pathways merits further research.

The question of why differential magnitudes of racial health disparities exist based on where one lives (rural/urban status) remains. Structural and systemic racism is pervasive in the United States. Still, little is known about how the levels of racism are both expressed and perceived comparing rural and urban areas and how that influences health and health behaviors across population subgroups. For example, do rural black populations experience greater perceived levels of stress or perhaps feel less safe to perform PA compared with urban black populations?^85,86^

Future research could provide insight as to what are the specific elements of race, racism, rural/urban status, and other related factors that drive these observed health disparities.

Furthermore, it is important to recognize that race is a social construct.^87^ The findings of this study in conjunction with findings of related studies^34,37–43^ using an intersectional framework to examine health disparities by race and rurality underscore the growing evidence that this social construct of race does not exercise its impact on population health uniformly across all populations. To identify and understand the important nuances in racial health disparities by rurality and potentially other place-based characteristics, research should focus on the health impacts of the intersectionality of social identities, including the critical intersections of how individuals perceive and internalize race and place-based factors.

Despite the vast body of research on black/white and rural/urban health disparities, the explanations for the results observed in this study that racial disparities in obesity and diabetes are more pronounced in rural areas than in the urban areas are less clear and merit further research. There is a small but growing body of research examining the potential for black/white health disparities to vary by rural/urban status and other factors related to place of residence.^78,88,89^

Still, the underlying causes for these variations remain unclear. It is also important to consider the heterogeneity among rural areas in research and policy development to improve health in rural areas and reduce rural/urban health disparities.^63,90,91^ Not all rural areas are alike in demographics, environmental factors, and social determinants of health.^92^ Therefore, interventions and policies aimed to reduce rural/urban disparities and reduce the magnitude of racial disparities in rural areas should consider and address the specific contextual factors in each rural region of interest to maximize the effectiveness of any such strategies.

### Limitations and strengths

The findings of this study should be interpreted in the context of several key limitations. First, this is a cross-sectional study, and so, causation cannot be inferred. Second, the main study objective was to assess how rural/urban status moderates the associations between race and obesity, one obesity-associated condition (diabetes), and health behavior (PA) using an intersectional framework. Obesity is a complex condition with numerous causes, correlations, and consequences that were not examined in this analysis. Similarly, race is a social construct and the current analysis was limited to adults who identified as black or white.

Other races and ethnicities were not examined in this study to explore the comparison between black and white respondents.^93^ Nonetheless, the sample included 76% of all BRFSS respondents. Next, all measures, including the outcomes of obesity, diabetes, and PA, as well as race and all covariates, were based on self-report data, which may induce systematic bias. For example, self-reported weight is likely to be lower than the actual weight, and the difference may be higher among women than men.^94^

Another limitation is that an unknown proportion of those who reported having diabetes may have had type 1 diabetes, which is caused by a nearly completely different set of risk factors.^95^ Although the analysis controlled for several important potential confounders, the findings are subject to residual confounding. Also of note, at the time of analysis, the data were ∼10 years old. The 2012 BRFSS data were used because it was the last year in which geographic identifiers (county of residence) were available in the database. Similarly, the analysis used area-level data on the county level. Counties are inherently heterogeneous, and the characteristics of a county, specifically the two measures of rural/urban status (IRR and RUCC), may not apply equally to all areas of each county and may vary by smaller geographic units within counties, such as census tract. Also, no geospatial analysis was conducted in this study.

Last, the only aspect of intersectionality examined in this study was race and rurality, which limits the collective understanding of the social structures and systems of disadvantage (e.g., gender, income, class) that contribute to obesity-related health outcomes, including PA.

This study had several notable strengths, however. First, this study is among the first to examine the potential for geographic or place-based characteristics—rural/urban status—to moderate well-documented racial health disparities in obesity and two obesity-associated measures using a large, nationally representative database. Second, the analysis considered rural/urban status as an ordinal variable, while many previous studies of rural/urban disparities considered the measure a dichotomous variable, which may mask more nuanced associations between rural/urban status and health outcomes.

Third, the analysis considered the possibility that the magnitude of the black/white disparities may be monotonic (Table 2) or nonmonotonic (Tables 3 and 4) with rural/urban status. In doing so, the analysis showed that the strength of the black/white disparities in all three outcome variables was highest in the second-highest quintile of rurality. Last, the analysis included a sensitivity analysis using a second measure of rural/urban status (RUCC) to determine if the associations observed were due to the way in which rural/urban status was measured.

## Conclusions

The results of this analysis found that black/white health disparities in obesity, diabetes, and PA persisted across all levels of rural/urban status. Still, the magnitude of these disparities and their prevalence tended to be higher in more rural areas than urban areas. Interventions and policies designed to improve obesity-related outcomes and reduce racial disparities should consider the geographic context in which these outcomes occur.

Furthermore, future studies should explore and address both the root causes of racial and rural/urban disparities, in which there is likely a substantial overlap using an intersectional lens. It is also critical to understand that race is a social construct, and the direct influences of race on health occur in multiple ways, including systemtic racism, poverty, culture, education, and numerous others. Such studies should also consider regional differences in these associations and how any trends extend to other types of health outcomes and behaviors beyond obesity.

## Abbreviations Used

BRFSS: Behavioral Risk Factor Surveillance System
CDC: Centers for Disease Control and Prevention
IRR: Index of Relative Rurality
PA: physical activity
Q1, Q2, etc.: quintile of rural/urban status (Q1=most rural, Q5=most urban)
RUCC: Rural/Urban Continuum Codes
SES: socioeconomic status

## References

[1] Braveman PA, Kumanyika S, Fielding J, et al. Health disparities and health equity: the issue is justice. Am J Public Health. 2011;101(S1):S149–S155. 21551385 10.2105/AJPH.2010.300062 PMC3222512

[2] National Center of Health Statistics (US). Health, United States, 2005: With Chartbook on Trends in the Health of Americans. Washington, DC: National Center on Health Statistics, Department of Health and Human Services, CDC, Department of Health and Human Services, Centers for Disease Control and Prevention, 2005, pp. 1–550.

[3] Woolf SH, Schoomaker H. Life expectancy and mortality rates in the United States, 1959–2017. JAMA. 2019;322:1996–2016. 31769830 10.1001/jama.2019.16932 PMC7146991

[4] Hunt BR, Hurlbert MS. Black: white disparities in breast cancer mortality in the 50 largest cities in the United States, 2005–2014. Cancer Epidemiol. 2016;45:169–173. 27720130 10.1016/j.canep.2016.07.018

[5] Yedjou CG, Sims JN, Miele L, et al. Health and racial disparity in breast cancer. Breast Cancer Metastasis Drug Resist. 2019:31–49. 10.1007/978-3-030-20301-6_3 PMC6941147 31456178

[6] Rosenstock S, Whitman S, West JF, et al. Racial disparities in diabetes mortality in the 50 most populous US cities. J Urban Health. 2014;91:873–885. 24532483 10.1007/s11524-013-9861-4 PMC4199450

[7] Weuve J, Barnes LL, de Leon CF, et al. Cognitive aging in black and white Americans: cognition, cognitive decline, and incidence of Alzheimer disease dementia. Epidemiol. 2018;29:151. 10.1097/EDE.0000000000000747 PMC5718953 28863046

[8] Whitson HE, Hastings SN, Landerman LR, et al. Black–white disparity in disability: the role of medical conditions. J Am Geriatr Soc. 2011;59:844–850. 21568956 10.1111/j.1532-5415.2011.03401.x PMC3107524

[9] Warner DF, Brown TH. Understanding how race/ethnicity and gender define age-trajectories of disability: an intersectionality approach. Soc Sci Med. 2011;72:1236–1248. 21470737 10.1016/j.socscimed.2011.02.034 PMC3087305

[10] Loggins S, Andrade FC. Despite an overall decline in US infant mortality rates, the Black/White disparity persists: recent trends and future projections. J Comm Health. 2014;39:118–123. 10.1007/s10900-013-9747-0 23929415

[11] Howell EA, Egorova N, Balbierz A, et al. Black-white differences in severe maternal morbidity and site of care. Am J Obstet Gyn. 2016;214:122.e1-7. 10.1016/j.ajog.2015.08.019 PMC4698019 26283457

[12] Leonard SA, Main EK, Scott KA, et al. Racial and ethnic disparities in severe maternal morbidity prevalence and trends. Ann Epidemiol. 2019;33:30–36. 30928320 10.1016/j.annepidem.2019.02.007 PMC6502679

[13] Dai D. Black residential segregation, disparities in spatial access to health care facilities, and late-stage breast cancer diagnosis in metropolitan Detroit. Health Place. 2010;16:1038–1052. 20630792 10.1016/j.healthplace.2010.06.012

[14] Andrasfay T, Goldman N. Reductions in 2020 US life expectancy due to COVID-19 and the disproportionate impact on the Black and Latino populations. Proc Natl Acad Sci USA. 2021;118–123. 10.1073/pnas.2014746118 PMC7865122 33446511

[15] Bull CN, Krout JA, Rathbone-McCuan E, et al. Access and issues of equity in remote/rural areas. J Rural Health. 2001;17:356–359. 12071561 10.1111/j.1748-0361.2001.tb00288.x

[16] Nelson JA, Stover Gingerich B. Rural health: access to care and services. Home Health Care Manage Pract. 2010;22:339–343.

[17] Cohen SA, Greaney ML, Sabik NJ. Assessment of dietary patterns, physical activity and obesity from a national survey: rural-urban health disparities in older adults. PLoS One. 2018;13:e0208268. 30517166 10.1371/journal.pone.0208268 PMC6281245

[18] O'Connor A, Wellenius G. Rural–urban disparities in the prevalence of diabetes and coronary heart disease. Public Health. 2012;126:813–820. 22922043 10.1016/j.puhe.2012.05.029

[19] Cohen SA, Cook SK, Kelley L, et al. A closer look at rural-urban health disparities: associations between obesity and rurality vary by geospatial and sociodemographic factors. J Rural Health. 2017;33:167–179. 27557442 10.1111/jrh.12207

[20] Weaver KE, Geiger AM, Lu L, et al. Rural-urban disparities in health status among US cancer survivors. Cancer. 2013;119:1050–1057. 23096263 10.1002/cncr.27840 PMC3679645

[21] Weeks WB, Kazis LE, Shen Y, et al. Differences in health-related quality of life in rural and urban veterans. Am J Public Health. 2004;94:1762–1767. 15451747 10.2105/ajph.94.10.1762 PMC1448531

[22] Singh GK, Siahpush M. Widening rural–urban disparities in all-cause mortality and mortality from major causes of death in the USA, 1969–2009. J Urban Health. 2014;91:272–292. 24366854 10.1007/s11524-013-9847-2 PMC3978153

[23] Singh GK, Azuine RE, Siahpush M, et al. All-cause and cause-specific mortality among US youth: socioeconomic and rural–urban disparities and international patterns. J Urban Health. 2013;90:388–405. 22772771 10.1007/s11524-012-9744-0 PMC3665977

[24] Zeng D, You W, Mills B, et al. A closer look at the rural-urban health disparities: insights from four major diseases in the Commonwealth of Virginia. Soc Sci Med. 2015;140:62–68. 26204561 10.1016/j.socscimed.2015.07.011

[25] Amaro H. The action is upstream: Place-based approaches for achieving population health and health equity. Am J Public Health. 2014;104:964. 24825190 10.2105/AJPH.2014.302032 PMC4062038

[26] Cosby AG, McDoom-Echebiri MM, James W, et al. Growth and persistence of place-based mortality in the United States: the rural mortality penalty. Am J Public Health. 2019;109:155–162. 30496008 10.2105/AJPH.2018.304787 PMC6301407

[27] Field AE, Coakley EH, Must A, et al. Impact of overweight on the risk of developing common chronic diseases during a 10-year period. Arch Intern Med. 2001;161:1581–1586. 11434789 10.1001/archinte.161.13.1581

[28] Francischetti EA, Genelhu VA. Obesity–hypertension: an ongoing pandemic. Intl J Clin Pract. 2007;61:269–280. 10.1111/j.1742-1241.2006.01262.x 17263714

[29] Rahmouni K, Correia ML, Haynes WG, et al. Obesity-associated hypertension: new insights into mechanisms. Hypertension. 2005;45:9–14. 15583075 10.1161/01.HYP.0000151325.83008.b4

[30] Sullivan PW, Ghushchyan VH, Ben-Joseph R. The impact of obesity on diabetes, hyperlipidemia and hypertension in the United States. Qual Life Res. 2008;17:1063–1071. 18777200 10.1007/s11136-008-9385-7

[31] Schienkiewitz A, Mensink GB, Scheidt-Nave C. Comorbidity of overweight and obesity in a nationally representative sample of German adults aged 18–79 years. BMC Public Health. 2012;12:1–1. 22894173 10.1186/1471-2458-12-658 PMC3526457

[32] Wormser D, Kaptoge S, Di Angelantonio E, et al. Emerging Risk Factors Collaboration. Separate and combined associations of body-mass index and abdominal adiposity with cardiovascular disease: collaborative analysis of 58 prospective studies. Lancet. 2011;377:1085–1095. 21397319 10.1016/S0140-6736(11)60105-0 PMC3145074

[33] Adams KF, Schatzkin A, Harris TB, et al. Overweight, obesity, and mortality in a large prospective cohort of persons 50 to 71 years old. New Engl J Med. 2006;355:763–778. 16926275 10.1056/NEJMoa055643

[34] Ogden CL, Fryar CD, Martin CB, et al. Trends in obesity prevalence by race and hispanic origin—1999–2000 to 20172018. JAMA. 2020;324:1208–1210. 32857101 10.1001/jama.2020.14590 PMC7455882

[35] Kim DD, Basu A. Estimating the medical care costs of obesity in the United States: systematic review, meta-analysis, and empirical analysis. Value Health. 2016;19:602–613. 27565277 10.1016/j.jval.2016.02.008

[36] Association AD. Economic costs of diabetes in the US in 2007. Diabetes Care. 2008;31:596–615. 18308683 10.2337/dc08-9017

[37] Rossen LM, Schoendorf KC. Measuring health disparities: trends in racial−ethnic and socioeconomic disparities in obesity among 2-to 18-year old youth in the United States, 2001–2010. Ann Epidemiol. 2012;22:698–704. 22884768 10.1016/j.annepidem.2012.07.005 PMC4669572

[38] May AL, Freedman D, Sherry B, et al., Centers for Disease Control and Prevention (CDC). Obesity—United States, 1999–2010. MMWR Surv Summ. 2013;62(Suppl 3):120–128. 24264501

[39] Zhang H, Rodriguez-Monguio R. Racial disparities in the risk of developing obesity-related diseases: a cross-sectional study. Ethnic Dis. 2012;22:308–316. 22870574

[40] Krueger PM, Reither EN. Mind the gap: race/ethnic and socioeconomic disparities in obesity. Curr Diab Rep. 2015;15:1–9. 10.1007/s11892-015-0666-6 PMC4947380 26377742

[41] Trivedi T, Liu J, Probst J, et al. Obesity and obesity-related behaviors among rural and urban adults in the USA. Rural Remote Health. 2015;15:3267. 26458564

[42] Wen M, Fan JX, Kowaleski-Jones L, et al. Rural–urban disparities in obesity prevalence among working age adults in the United States: exploring the mechanisms. Am J Health Prom. 2018;32:400–408. 10.1177/0890117116689488 29214811

[43] Befort CA, Nazir N, Perri MG. Prevalence of obesity among adults from rural and urban areas of the United States: findings from NHANES (2005-2008). J Rural Health. 2012;28:392–397. 23083085 10.1111/j.1748-0361.2012.00411.x PMC3481194

[44] LaVeist T, Pollack K, Thorpe Jr R, et al. Place, not race: disparities dissipate in southwest Baltimore when blacks and whites live under similar conditions. Health Affair. 2011;30:1880–1887. 10.1377/hlthaff.2011.0640 PMC6535343 21976330

[45] Bowleg L. The problem with the phrase women and minorities: intersectionality—an important theoretical framework for public health. Am J Public Health. 2012;102:1267–1273. 22594719 10.2105/AJPH.2012.300750 PMC3477987

[46] Abrams JA, Tabaac A, Jung S, et al. Considerations for employing intersectionality in qualitative health research. Soc Sci Med. 2020;258:113138. 32574889 10.1016/j.socscimed.2020.113138 PMC7363589

[47] Hopkins P. Social geography I: intersectionality. Prog Hum Geogr. 2019;43:937–947.

[48] Richman L, Pearson J, Beasley C, et al. Addressing health inequalities in diverse, rural communities: an unmet need. SSM-Pop Health. 2019;7:100398. 10.1016/j.ssmph.2019.100398 PMC6462771 31011618

[49] Erwin PC, Fitzhugh EC, Brown KC, et al. Health disparities in rural areas: the interaction of race, socioeconomic status, and geography. J Health Care Poor Undeserved. 2010;21:931–945. 10.1353/hpu.0.0336 20693736

[50] Centers for Disease Control and Prevention. Behavioral Risk Factor Surveillance System 2012 Codebook Report: Landline and Cell-Phone Data. Available at https://www.cdc.gov/brfss/annual_data/2012/pdf/CODEBOOK12_LLCP.pdf Accessed September 26, 2021.

[51] Centers for Disease Control and Prevention. Behavioral Risk Factor Surveillance System 2012 Overview. Available at https://www.cdc.gov/brfss/annual_data/2012/pdf/Overview_2012.pdf Accessed September 26, 2021.

[52] Hart CL, Morrison DS, Batty GD, et al. Effect of body mass index and alcohol consumption on liver disease: analysis of data from two prospective cohort studies. BMJ. 2010;340:c1240. 20223873 10.1136/bmj.c1240 PMC2837144

[53] Waldorf BS. A continuous multi-dimensional measure of rurality: moving beyond threshold measures. American Agricultural Economics Association, 2006. Available at https://ageconsearch.umn.edu/record/21383 Accessed August 2, 2021.

[54] Cossman RE, Cossman JS, Cosby AG, et al. Reconsidering the rural–urban continuum in rural health research: a test of stable relationships using mortality as a health measure. Popul Res Policy Rev. 2008;27:459–476.

[55] Cohen SA, Kelley L, Bell AE. Spatiotemporal discordance in five common measures of rurality for US counties and applications for health disparities research in older adults. Front Public Health. 2015;3:267. 26636064 10.3389/fpubh.2015.00267 PMC4658471

[56] Segel JE, Lengerich EJ. Rural-urban differences in the association between individual, facility, and clinical characteristics and travel time for cancer treatment. BMC Public Health. 2020;20:1–0. 32028942 10.1186/s12889-020-8282-z PMC7006189

[57] Probst JC, Zahnd WE, Hung P, et al. Rural-urban mortality disparities: variations across causes of death and race/ethnicity, 2013–2017. Am J Public Health. 2020;110:1325–1327. 32673111 10.2105/AJPH.2020.305703 PMC7427230

[58] Zahnd WE, Murphy C, Knoll M, et al. The intersection of rural residence and minority race/ethnicity in cancer disparities in the United States. Intl J Environ Res Public Health. 2021;18:1384. 10.3390/ijerph18041384 PMC7913122 33546168

[59] Dahly DL, Adair LS. Quantifying the urban environment: a scale measure of urbanicity outperforms the urban–rural dichotomy. Soc Sci Med. 2007;64:1407–1419. 17196724 10.1016/j.socscimed.2006.11.019 PMC2001275

[60] Li Y, Wang Y, Morrow-Howell N. Neighborhood effects on the health of Chinese older adults: beyond the rural and urban dichotomy. Gerontologist. 2021;61:403–412. 32598467 10.1093/geront/gnaa081

[61] Isserman AM. In the national interest: defining rural and urban correctly in research and public policy. Int Regional Sci Rev. 2005;28:465–499.

[62] Schaeffer PV, Kahsai MS, Jackson RW. Beyond the rural–urban dichotomy: essay in honor of Professor AM Isserman. Int Regional Sci Rev. 2013;36:81–96.

[63] Waldorf B, Kim A. Defining and measuring rurality in the US: from typologies to continuous indices. In: Commissioned Paper Presented at the Workshop on Rationalizing Rural Area Classifications, Washington, DC: Keck Center, 2015.

[64] Williams DR, Rucker TD. Understanding and addressing racial disparities in health care. Health Care Fin Rev. 2000;21:75. PMC4194634 11481746

[65] Yearby R. Racial disparities in health status and access to healthcare: the continuation of inequality in the United States due to structural racism. Am J Econ Soc. 2018;77:1113–1152.

[66] Neblett Jr EW. Racism and health: challenges and future directions in behavioral and psychological research. Cult Diver Ethn Min. 2019;25:12. 10.1037/cdp0000253 30714763

[67] Williams DR, Sternthal M. Understanding racial-ethnic disparities in health: sociological contributions. J Health Soc Behav. 2010;51(1_ suppl):S15–S27. 20943580 10.1177/0022146510383838 PMC3468327

[68] Brondolo E, Gallo LC, Myers HF. Race, racism and health: disparities, mechanisms, and interventions. J Behav Med. 2009;32:1–8. 19089605 10.1007/s10865-008-9190-3

[69] Williams DR, Mohammed SA. Discrimination and racial disparities in health: evidence and needed research. J Behav Med. 2009;32:20–47. 19030981 10.1007/s10865-008-9185-0 PMC2821669

[70] Lockwood KG, Marsland AL, Matthews KA, et al. Perceived discrimination and cardiovascular health disparities: a multisystem review and health neuroscience perspective. Ann NY Acad Sci. 2018;1428:170–207. 30088665 10.1111/nyas.13939

[71] Hall WJ, Chapman MV, Lee KM, et al. Implicit racial/ethnic bias among health care professionals and its influence on health care outcomes: a systematic review. Am J Public Health. 2015;105:e60–e76. 10.2105/AJPH.2015.302903 PMC4638275 26469668

[72] Williams DR, Collins C. Racial residential segregation: A fundamental cause of racial disparities in health. Public Health Rep. 2001;116:404–416. 12042604 10.1093/phr/116.5.404 PMC1497358

[73] White K, Borrell LN. Racial/ethnic residential segregation: framing the context of health risk and health disparities. Health Place. 2011;17:438–448. 21236721 10.1016/j.healthplace.2010.12.002 PMC3056936

[74] Robert SA, Ruel E. Racial segregation and health disparities between black and white older adults. J Gerontol B Psych Sci Soc Sci. 2006;61:S203–S211. 10.1093/geronb/61.4.s203 16855041

[75] Koh HK, Oppenheimer SC, Massin-Short SB, et al. Translating research evidence into practice to reduce health disparities: a social determinants approach. Am J Public Health. 2010;100(S1):S72–S80. 20147686 10.2105/AJPH.2009.167353 PMC2837437

[76] Byun SY, Meece JL, Irvin MJ. Rural-nonrural disparities in postsecondary educational attainment revisited. Am Educ Res J. 2012;49:412–437. 10.3102/0002831211416344 PMC3839859 24285873

[77] Welch A, Helme S, Lamb S. Rurality and inequality in education. In: International Studies in Educational Inequality, Theory and Policy. Edited by Teese R, Lamb S, Duru-Bellat M, Helme S. Dordrecht: Springer, 2007, pp. 602–624.

[78] Probst JC, Moore CG, Glover SH, et al. Person and place: the compounding effects of race/ethnicity and rurality on health. Am J Public Health. 2004;94:1695–1703. 15451735 10.2105/ajph.94.10.1695 PMC1448519

[79] Leider JP, Meit M, McCullough JM, et al. The state of rural public health: enduring needs in a new decade. Am J Public Health. 2020;110:1283–1290. 32673103 10.2105/AJPH.2020.305728 PMC7427223

[80] Heflin C, Miller K. The geography of need: identifying human service needs in rural America. J Fam Soc Work. 2012;15:359–374.

[81] Basu S, Berkowitz SA, Phillips RL, et al. Association of primary care physician supply with population mortality in the United States, 2005-2015. JAMA Int Med. 2019;179:506–514. 10.1001/jamainternmed.2018.7624 PMC6450307 30776056

[82] Tran P, Tran L, Tran L. Impact of rurality on diabetes screening in the US. BMC Public Health. 2019;19:1–0. 31554513 10.1186/s12889-019-7491-9 PMC6761709

[83] Callaghan T, Lueck JA, Trujillo KL, et al. Rural and urban differences in COVID-19 prevention behaviors. J Rural Health. 2021;37:287–295. 33619836 10.1111/jrh.12556 PMC8013340

[84] Parks SE, Housemann RA, Brownson RC. Differential correlates of physical activity in urban and rural adults of various socioeconomic backgrounds in the United States. J Epidemiol Comm Health. 2003;57:29–35. 10.1136/jech.57.1.29 PMC1732269 12490645

[85] Mujahid MS, Gao X, Tabb LP, et al. Historical redlining and cardiovascular health: The Multi-Ethnic Study of Atherosclerosis. Proc Natl Acad Sci U S A. 2021;118:e2110986118. 34903653 10.1073/pnas.2110986118 PMC8713797

[86] Simons RL, Lei MK, Klopack E, et al. The effects of social adversity, discrimination, and health risk behaviors on the accelerated aging of African Americans: further support for the weathering hypothesis. Soc Sci Med. 2021;282:113169. 32690336 10.1016/j.socscimed.2020.113169 PMC7790841

[87] Bryant BE, Jordan A, Clark US. Race as a social construct in psychiatry research and practice. JAMA Psychiatry. 2021 (In press). DOI :10.1001/jamapsychiatry.2021.2877. PMC10519151 34878501

[88] Johnson KS, Kuchibhatla M, Payne R, et al. Race and residence: intercounty variation in black-white differences in hospice use. J Pain Symptom Manage. 2013;46:681–690. 23522516 10.1016/j.jpainsymman.2012.12.006 PMC3735723

[89] Hall JE, Moonesinghe R, Bouye K, et al. Racial/ethnic disparities in mortality: contributions and variations by rurality in the United States, 2012–2015. Intl J Environ Res Public Health. 2019;16:436. 10.3390/ijerph16030436 PMC6388242 30717345

[90] Rigg KK, Monnat SM, Chavez MN. Opioid-related mortality in rural America: geographic heterogeneity and intervention strategies. Intl J Drug Policy. 2018;57:119–129. 10.1016/j.drugpo.2018.04.011 29754032

[91] National Academies of Sciences, Engineering, and Medicine. Rationalizing Rural Area Classifications for the Economic Research Service: A Workshop Summary. Washington, DC: National Academies Press, 2016.

[92] Mudd-Martin G, Biddle MJ, Chung ML, et al. Rural Appalachian perspectives on heart health: social ecological contexts. Am J Health Behav. 2014;38:134–143. 24034688 10.5993/AJHB.38.1.14

[93] Kravitz-Wirtz N. Temporal effects of child and adolescent exposure to neighborhood disadvantage on black/white disparities in young adult obesity. J Adolesc Health. 2016;58:551–557. 26995292 10.1016/j.jadohealth.2016.01.004 PMC4842347

[94] Niedhammer I, Bugel I, Bonenfant S, et al. Validity of self-reported weight and height in the French GAZEL cohort. Intl J Obes Relat Metab Disord. 2000;24:1111–1118. 10.1038/sj.ijo.0801375 11033979

[95] Zaccardi F, Webb DR, Yates T, et al. Pathophysiology of type 1 and type 2 diabetes mellitus: a 90-year perspective. Postgrad Med J. 2016;92:63–69. 26621825 10.1136/postgradmedj-2015-133281

